# Allelic frequency of *msp*2 and *glurp* genes in *Plasmodium falciparum* isolates from Awka, Anambra, Nigeria

**DOI:** 10.5281/zenodo.14886922

**Published:** 2025-02-18

**Authors:** Moses Ikegbunam, Abone Harrison, Chukwudi Egbuche, Nwasolu Obidi, Judith Mbamalu, Enyi Emmanuel, Offojebe Kosisochukwu, Mercy Ezeunala, Nzeukwu Chibumma, Ifeyinwa Onochie-Igbinedion, Joy Igwe, Joy Nnanna, Dorothy Ezeagwuna, Vincent Duru, Frances Nworji, Charles Esimone

**Affiliations:** 1 Department of Pharmaceutical Microbiology and Biotechnology, Nnamdi Azikiwe University, Awka Nigeria.; 2 Molecular Research foundation for students and scientists, Nnamdi Azikiwe University, Awka, Nigeria.; 3 National Institute for Pharmaceutical Research and development (NIPRD), Idu, Abuja, Nigeria.; 4 Department of Parasitology and Entomology, Nnamdi Azikiwe University, Awka, Nigeria.; 5 Department of Pharmaceutical microbiology and Biotechnology, Abia state University, Uturu, Abia State.; 6 Department of Biological Sciences (Microbiology Unit), Dennis Osadabay University, Asaba Delta State.; 7 Department of Applied Biochemistry, Nnamdi Azikiwe University, Awka, Nigeria.

## Abstract

**Introduction:**

The genetic diversity of *Plasmodium falciparum* correlates with its pathogenicity, therefore design of evidence-based intervention strategies to eradicate malaria requires genetic diversity surveillance. This study characterised the allelic frequencies and genetic diversity of *P. falciparum* parasites isolated from Awka, Nigeria.

**Materials and Methods:**

Genomic DNA was extracted from 177 *P. falciparum* isolates and the polymorphic regions of the *msp2* and *glurp* genes were genotyped by nested polymerase chain reaction (PCR).

**Results:**

Two *msp2* alleles (3D7 and FC27) were analysed. The 3D7 (93.55%) *msp2* allelic family was predominant in *msp2* positivie isolates. Polyclonal *msp2* infection was observed in 24 (38.71%) isolates. Twenty-one distinct *msp2* alleles were detected, with fragment sizes ranging from 200 bp to 1200 bp. The 300 bp allelic fragment (26.83%) was predominant for the 3D7 allele, while the 400 bp allelic fragment (29.54%) was predominant for the FC27 allele. The multiplicity of infection (MoI) in *msp2* was 2.03, and the expected Heterozygosity (He) was 0.34. Sixty-nine isolates (38.98%) were positive for the RII repeat region of the *glurp* gene. For the *glurp* gene, nine alleles were detected for fragment sizes ranging from 200 bp to 1150 bp, and the most prevalent allelic fragment was 200 bp (19%). The MoI and He for the *glurp* gene were 0.45 and 0.98, respectively.

**Conclusions:**

The high level of polyclonal infections with *P. falciparum* parasites observed in this study indicates extensive genetic diversity in the study area. The data provide important baseline information that can be implemented in developing malaria control strategies and elimination in the study area and Nigeria.

## Introduction

Nigeria has the highest burden of malaria globally. It is a major public health concern in Nigeria with about 200,000 deaths from the disease annually, the main victims being children <5 yrs and pregnant women [1]. *Plasmodium falciparum* is the deadliest and most prevalent plasmodium species associated with malaria infection in sub-Saharan Africa [1,2]. The extensive genetic diversity of *P. falciparum* strains poses a challenge to efforts to eradicate malaria and possibly contributes to malaria pathology by suppressing acquired immunity and prompting the emergence of drug resistance and insecticide resistance variants [3,4,5]. Therefore, genomic surveillance of the parasite is essential to develop an effective control strategy to eradicate malaria in Nigeria.

An established method to survey the parasite is by targeted genotyping of merozoite surface protein (*msp-2)* and glutamate-rich protein (*glurp*) genes as they contain polymorphic regions that are used as markers for defining genetic variation in *P. falciparum* malaria and determining the multiplicity of infection (MoI) [4,6,7]. The *msp-2* glycoprotein is an asexual blood stage antigen that consists of 5 polymorphic blocks with block 3 being the most polymorphic. The two main allelic families of *msp-2*, FC27 and 3D7, are based on the polymorphic regions of the central repeat sequences [8,9]. The *glurp* protein is an antigen expressed in both the pre-erythrocytic and erythrocytic stages of the parasite, as well as on the surface of newly released merozoites. This antigen consists of three regions, with the immunodominant C-terminal repetitive region (R2) being the most polymorphic [10]. The *msp-2* and *glurp* proteins are also considered promising candidate antigens for the development of a malaria vaccine as they are targeted by cytophilic antibodies and are associated with natural immune protection against clinical malaria [11,12],

Understanding the genetic diversity of *P. falciparum* in different geographical regions of Nigeria is essential for developing new and effective malaria control interventions. Currently, there is limited information on the genetic diversity and multiplicity of *P. falciparum* infection in southeast Nigeria. This study aimed to evaluate the allelic frequency of *msp2* and *glurp* genes in *P. falciparum* parasites isolated in Awka, an urban city in southeast Nigeria. This research will contribute information on disease pathogenesis and immunity acquisition in Nigeria. It also provides information that is benefcial for the development of an effective malaria vaccine.

## Materials and Methods

This survey was conducted to assess the allelic frequency of *P. falciparum msp2* and *glurp* genes. A total of 179 participants, aged 6 months to 68 yrs, were recruited between February 2019 and January 2020. Participants were included if they presented with malaria-related symptoms (e.g., fever, chills, headache) at Chukwuemeka Odumegwu Ojukwu University Teaching Hospital, Awka, Anambra State, Nigeria. The recruitment was based on clinical suspicion of malaria, and participants were screened using a rapid diagnostic test (RDT) for malaria prior to enrolment. Written informed consent was obtained from all participants or their legal guardians before inclusion in the study.

### Study site and population

The study was conducted in Awka, Anambra State, Nigeria, a region with a tropical climate characterised by a wet season from April to October and a dry season from November to March. The area experiences an average annual rainfall of 1,200 mm and a temperature range of 25–32°C. Malaria transmission in this region is perennial, with peaks during the rainy season. Control measures include the use of insecticide-treated nets (ITNs) and intermittent preventive treatment for pregnant women (IPTp).

Participants were recruited from outpatient clinics, with inclusion criteria as follows: Age between 4 months and 60 years, presentation with clinical signs and symptoms suggestive of malaria and positive RDT for malaria prior to study enrolment. Exclusion criteria included participants on malaria treatment within the last two weeks or those unwilling to provide informed consent.

### Sample collection, preparation and parasite detection

Venous blood samples (2 mL) were collected into EDTA tubes from each participant. RDT was performed using the SD Bioline Malaria Ag Pf/Pan (South Korea), which detects *P. falciparum* and non-falciparum species. Samples positive by RDT were preserved at −20°C until genomic DNA extraction. DNA extraction was performed using the Quick-DNA Miniprep kit (Zymo Research, USA) following the manufacturer’s protocol.

### Molecular genotyping of *P. falciparum msp2* and *glurp* genes

The polymorphic regions of the *msp2* and *glurp* genes were amplified in a nested PCR reaction. The primary PCR conditions for both the *msp2* and *glurp* genes were an initial denaturation step of 95 °C for 5 min followed by 30 cycles of 95 °C for 1 min, 54 °C for 1 min, 72 °C for 1 min, and a final extension of 72 °C for 5 min. The nested PCR parameters for the *glurp* gene were identical to the primary reaction; only the annealing temperature was adjusted to 59 °C. For the *msp2* gene, the nested PCR conditions were initial denaturation at 94°C for 5 min, followed by 30 cycles at 94 °C for 10 s, 57 °C for 30 s, and 72 °C for 40 s. The final cycle had a prolonged extension at 72 °C for 3 min. The primers targeting the RII region of *glurp* [13] and the 3D7 and FC27 regions of *msp2* [14] are shown in Table 1. The PCR products were separated on a 1.5% agarose gel, stained with ethidium bromide, and visualised using a UV transilluminator (Vilber, France).

**Table 1:** Different sequences of the primers used to amplify *msp2* and *glurp* genes of *P. falciparum* isolates.

Gene	Primers
***msp2* (N1)**	F: 5'-ATGAAGGTAATTAAAACATTGTCTATTATA-3'
	R: 5'-TTATATGAATATGGCAAAAGATAAAACAAG-3'
**FC27 family (N2)**	F: 5'-GCAAATGAAGGTTCTAATACTAATAG-3'
	R: 5'-GCTTTGGGTCCTTCTTCAGTTGATTC-3'
**3D7 family (N2)**	F: 5'-GCAGAAAGTAAGCCTCTACTGGTGCT-3'
	F: 5'-GATTGTTTCGGCATTATTATGA-3'
***glurp* (N1)**	F: 5'-TGAATTTGAAGATGTTCACACTGAAC-3'
	R1: 5'-ACATGCAAGTGTTGATCCTGAAG-3'
**(N2)**	F: 5'-TGTTCACACTGAACAATTAGATTTAGATCA-3'
	R2: 5'-TGTAGGTACCACGGGTTCTTGTGG-3'

### Heterozygosity and multiplicity of infection

The expected heterozygosity index (He) was calculated using the formula:

He=[nn−1][1−∑Pi2]

Where *n* = number of isolates analysed and *Pi* = the frequency of the *i*^th^ allele in the population. The multiplicity of infection (MoI) was calculated by dividing the total number of fragments detected in an antigenic marker by the number of samples positive for that same marker. Samples where only one genotype was detected per allelic family were monoclonal, while samples with two or more genotypes per allelic family were polyclonal *P. falciparum* infections.

### Data analysis

Heterozygosity index (He) and multiplicity of infection (MoI) were calculated. The presence of polyclonal infections was determined based on the detection of multiple alleles within the same sample.

### Ethical approval

The Chukwuemeka Odumegwu Ojukwu University Teaching Hospital's Ethics Review Board granted ethical approval for the study. (COOUTH/CMAC/ETH.C/Vol.1/0035). Informed consent was obtained from the parent or legal guardian of each child before being included in the study.

## Results

The study population consisted of 53.03% female and 46.97% male patients aged 6 months to 68 years The mean age of participants was 18.67 ± 0.32 yrs. A total of 179 blood samples were analysed for *Plasmodium* spp. using a malaria RDT kit which confirmed the presence of *P. falciparum* in 177 (99%) samples.

### Genetic diversity of *P. falciparum* infection

**Figure 1:**
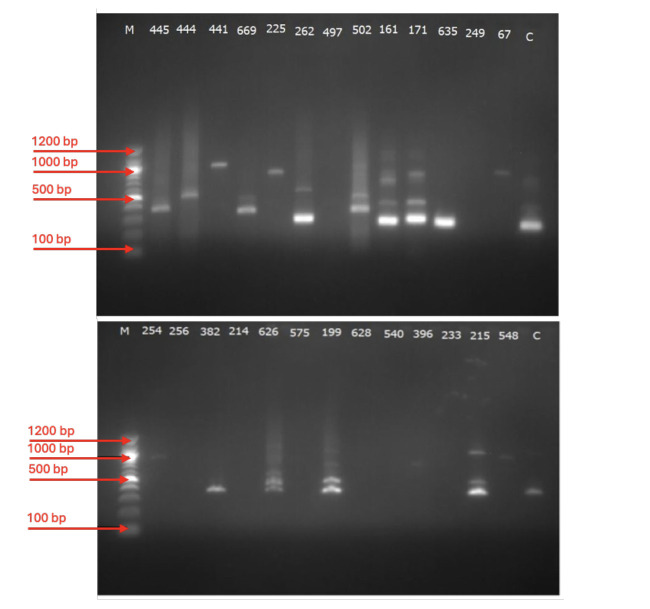
Representative Gel images of samples showing different alleles for 3D7 (top) and FC27 (bottom).

PCR amplification was successful in 35.02% (62/177) of the isolates for the *msp2* gene and 38.98% (69/177) of the isolates for the *glurp* gene (Table 2). For *msp2*, the Fc27 allele had a frequency of 45.16% (28/62) while the predominant 3D7 allele had a frequency of 93.55% (58/62). Monoclonal infections were identified in 38 isolates (61.29%), with 34 isolates (54.84%) positive for the 3D7 allele and 4 isolates (6.45%) positive for the FC27 allele. Polyclonal infections (FC27+IC3D7) were detected in 24 isolates (38.71%). The RII repeat region of the *glurp* gene was detected in all 69 isolates. Representative gel pictures are presented in Figures 1.

**Table 2:** Prevalence of *msp2* and *glurp* genes.

Markers	Frequency (%)	Allele size (bp)	Total No of Alleles	Distinct alleles
**FC27**	28 (45.16%)	250 -1200	44	10
**3D7**	58 (93.55%)	200 -1100	82	11
**FC27 + 3D7**	24 (38.71%)			
***Glurp***	69 (38.98%)	200-1150	79	9

### Allelic frequency of *msp2* and *glurp* genes

**Figure 2:**
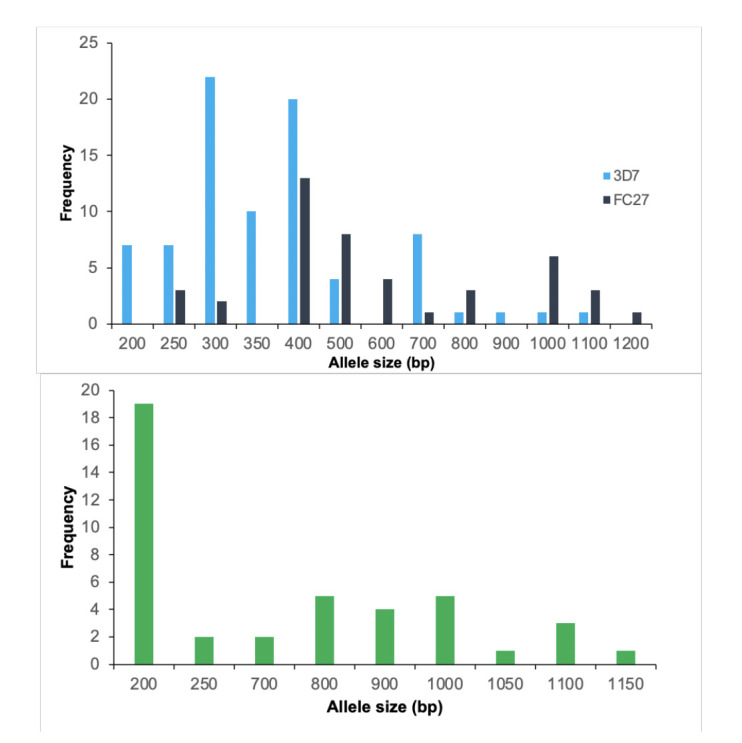
Prevalence of *P*. *falciparum* 3D7 and FC27 *msp2* alleles (top) and *glurp* alleles (bottom).

The allelic genotyping data revealed the polymorphic nature of *P. falciparum* parasites in Awka. In the *msp2* and *glurp* genes, different allelic types were identified. Alleles of *msp2* and *glurp* were classified according to the size of the amplified PCR bands. Twenty-one alleles were detected in *P. falciparum* isolates positive for the *msp2* gene. This includes 10 FC27 alleles and 11 3D7 alleles with 200–1200 bp fragment sizes. The 300 bp allelic fragment (26.83%) was predominant for the 3D7 allele, while the 400 bp allelic fragment (29.55%) was predominant for the FC27 allele (Figure 2). PCR amplification for the RII repeat region of the *glurp* gene was successful in 69 isolates. Nine alleles with fragment sizes ranging from 200 to 1150 bp were identified. The most prevalent allelic fragment was 200 bp (45.24%), while both the 1050 bp and 1150 bp allelic fragments were the least prevalent (2.38%) (Figure 2).

### Multiplicity of infection (MoI)

The MoI for the *msp2* gene (MoI = 2.03) was high as was the heterozygosity (He = 0.34). The MoI and He for the *glurp* gene were 0.45 and 0.98, respectively.

## Discussion

Nigeria is a malaria-endemic country, and an increase in the genetic diversity of *P. falciparum* strains could lead to more complex infections and the emergence of more virulent or drug-resistant variants, endangering efforts to eradicate the disease [15]. Genetic diversity and polymorphism are key in the acquisition of anti-malaria parasite immunity [16,17]. Therefore, determining the frequency of *Plasmodium* genotypes in different geographical locations would facilitate the development of effective control strategies. Polymorphic markers in *P. falciparum* isolates were used to examine the genetic diversity and complexity of parasite populations in patients with symptomatic malaria at the Chukwuemeka Odumegwu Ojukwu Teaching Hospital, Awka, Nigeria.

In this study, allele-specific genotyping of *msp2* in *P. falciparum* isolates reveals high allelic diversity in Awka, Nigeria. The high malaria transmission rate, the incidence of mixed illnesses, and the subsequent exposure of locals to mosquito bites may be responsible for this trend in the research area. For *msp2*, the 3D7 alleles were predominant, with a 93.54% occurrence. This data is consistent with studies from Kaduna [18], Ibadan [19], Anambra [7], and north-central Nigeria [20, 21] that reported a high prevalence of the 3D7 family, in symptomatic patients, but the results contrast with a study by Ojurongbe *et al.* [22] in Osogbo, Nigeria, which reported the FC27 allele as the predominant allele. The result is in agreement with other studies conducted in sub-Saharan Africa [23,24], South America [16], and Asia [17,25]. The results suggest that the frequency of 3D7 alleles strongly correlates with symptomatic malaria. The result is conflicting as the 3D7 allele is associated with asymptomatic malaria infections, and it is thought to offer protection against clinical disease [26]. The contrasting observation from various studies indicates a need to understand the influence of human genetic factors on the antigenicity of *msp2* alleles as the variations observed in different studies may be attributed to immune selection pressures.

Among the *msp2* positive *P. falciparum* isolates, polyclonal infections were observed in 38.71% of participants. Complex infections marked by multiclonality impacts drug efficacy, severity of disease and the population diversity of the parasite [4]. Polyclonal infections are associated with low levels of protective malaria antibodies, increased prevalence of drug-resistant parasites and possibility of recrudescence [27,28]. More importantly, malaria vaccine efficacy studies on the *msp2* gene have shown that vaccination with only one *msp2* variant induces an allele-specific response. In one study involving a vaccine that comprised the 3D7 allelic family, vaccinated patients showed an increase in morbidity associated with the FC27 alleles [29]. Subsequent studies have shown that chimeric vaccines [30] and vaccines containing both *msp2* allelic families [31] induce a strain-transcending immune response. This necessitates that the malaria vaccine design incorporates both allelic variants of the *msp2* gene to account for the increase in polyclonal infections observed in this study.

The high genetic diversity for *msp2* is consistent with observations in malaria-endemic regions [4]. The high genetic diversity may correlate with the rate of polyclonal infections and the intensity of malaria transmission in the region [4]. The multiplicity of *P. falciparum* infections (MoI) for *msp2* reported in this study is similar to studies from Nnewi [7] and Ibadan [32] in Nigeria. However, other studies from southwest Nigeria [33,34,35] and Pahang, Malaysia [36] reported a lower MoI, while a study on children living close to a lake in Taabo, Côte d’Ivoire, reported a higher MoI. The multiplicity of infection (MoI), i.e., the number of different *P. falciparum* strains co-infecting a single host in many malaria-endemic areas is a common feature and has been reported to vary with age, parasite density, immune status, epidemiological settings, and transmission intensity [37]. The MoI observed in this study could indicate high transmission levels of the parasite.

The RII region of the *glurp* was also shown to have a high prevalence in the study population, with 38.98% occurrence. The study by Ullah *et al.* [6] showed a higher prevalence of 70% in Pakistan, consistent with another study conducted in south-western Nigeria [32]. Furthermore, a study in Osogbo, Nigeria, reported that the genetic diversity of the R2 polymorphic region of the *glurp* gene remained diverse despite the implementation of the artemisinin-based combination therapy ACT therapy in the study area [38]. The *glurp* gene is an antigen of *P. falciparum* that is highly conserved, present in all stages of the malaria parasite and associated with clinical immunity. These attributes make it a promising biomarker for diagnosis and the development of vaccines against malaria.

## Conclusions

The present study shows that there is a high level of polyclonal *P. falciparum* infections in the population. The *P. falciparum* parasites harbour multiple gene alleles with high MoI. This indicates the extensive genetic diversity of *P. falciparum* infection in the study area. The data provides important baseline information to guide malaria control and elimination strategies in the study area and across Nigeria.
